# Transcriptome profiling of macrophages persistently infected with human respiratory syncytial virus and effect of recombinant *Taenia solium* calreticulin on immune-related genes

**DOI:** 10.3389/fmicb.2024.1402589

**Published:** 2024-09-04

**Authors:** Evelyn Rivera-Toledo, Miguel A. Fernández-Rojas, Carlos Santiago-Olivares, Mayra Cruz-Rivera, Vania Hernández-Bautista, Fernanda Ávila-Horta, Ana Flisser, Fela Mendlovic

**Affiliations:** ^1^Departamento de Microbiología y Parasitología, Facultad de Medicina, Universidad Nacional Autónoma de México (UNAM), Mexico City, Mexico; ^2^Facultad de Ciencias de la Salud, Universidad Anáhuac México Norte, Huixquilucan de Degollado, Mexico

**Keywords:** P388D1 cell line, viral persistence, antiviral activity, calreticulin, HRSV

## Abstract

**Introduction:**

Human respiratory syncytial virus (hRSV) is a main cause of bronchiolitis in infants and its persistence has been described in immunocompromised subjects. However, limited evidence has been reported on the gene expression triggered by the hRSV and the effect of recombinant *Taenia solium*-derived calreticulin (rTsCRT).

**Methods:**

Using a comprehensive microarray approach, we analyzed the transcriptome profile of a macrophage cell line that has supported hRSV persistence for over 150 passages. We compared the gene expression of persistently infected and non-infected macrophages. We also evaluated the effect of rTsCRT on hRSV-infected macrophage gene transcription, as well as on cytokine production and number of copies of the persistent hRSV genome.

**Results:**

Our analysis showed that hRSV long-term virus infection significantly alters mRNA expression of antiviral, inflammatory, as well as arginine and lipid metabolism-associated genes, revealing a transcriptional signature that suggests a mixed M1/M2 phenotype. The resulting host-virus equilibrium allows for the regulation of viral replication, while evading the antiviral and proinflammatory responses. Interestingly, rTsCRT stimulus upregulated *Tnfα*, *Il6* and *Nos2* mRNA. We found increased levels of both proinflammatory cytokines and nitrite levels in the conditioned media of persistent macrophages treated with rTsCRT. This increase was associated with a significant reduction in viral genome copies.

**Discussion:**

hRSV persistently infected macrophages retain responsiveness to external stimuli and demonstrate that the profound changes induced by viral persistence are potentially reversible. Our observations contribute to the understanding of the mechanisms related to hRSV persistence in macrophages and have implications for the development of targeted therapies to eliminate persistent infections or reduce the negative effects related with chronic inflammatory diseases associated with hRSV infection.

## 1 Introduction

Human respiratory syncytial virus (hRSV) is a leading cause of bronchiolitis and pneumonia in infants and is linked to the development of airway hyperreactivity in children who suffer severe disease early in life (Rosas-Salazar et al., 2023). hRSV infection in the elderly is also of concern, as the incidence of mortality may exceed that estimated for infants by a factor of 20 (Coultas et al., 2019).

hRSV is an enveloped virus belonging to the *Pneumoviridae* family, Orthopneumovirus genus. It possesses a single-stranded negative sense RNA genome and contains 10 genes that encode for 11 proteins (Battles and McLellan, 2019). Viral PAMPs (pathogen associated molecular patterns), like proteins and single-stranded or double-stranded RNA (ssRNA or dsRNA) trigger signaling pathways that activate transcription factors such as interferon regulatory factors (IRFs), NF-κB, ATF2 and c-Jun to establish an antiviral and proinflammatory state (Kawai and Akira, 2006; Carty et al., 2021). Particularly, hRSV nucleic acids are recognized through RLRs (ddx58/RIG-I and Ifih1/MDA5), TLR3, TLR7 and TLR8, while proteins are detected by TLR2, TLR6 and TLR4 (Ouyang et al., 2022).

Proinflammatory cytokines and chemokines that recruit leukocytes are produced during hRSV infection and contribute to viral clearance. However, in younger infants hRSV, severe disease is associated with a deregulated inflammatory response associated with the development of recurrent wheezing and asthma-like symptoms (Roe et al., 2011; Russell et al., 2017). The mechanisms linked to these respiratory complications are not clear. However, they appear to be multifactorial, involving factors such as age, host-genetic predisposition, immune responses, and the virus’s ability to establish persistent infections (Bertrand et al., 2015; Bont and Ramilo, 2011; Kato et al., 2012; Mejías et al., 2005; Piedimonte and Perez, 2014; Wu and Hartert, 2011).

hRSV persistence has been described in the respiratory tract of immunocompromised subjects and in mouse models (Tabatabai et al., 2018). hRSV genomic RNA and viral messenger RNA were detected for over 100 days after primary infection in mice. Recovery of infectious virus occurred only after depletion of CD4 and CD8 T lymphocytes, suggesting that the immune response has an essential role in viral elimination (Schwarze et al., 2004). Furthermore, hRSV persistence has been established in human epithelial cell lines and mouse macrophages (Martínez et al., 2009; Sarmiento et al., 2002; Valdovinos and Gómez, 2003). Cell lines are a valuable tool to characterize the virus-host cell interactions responsible for the establishment and maintenance of long-term infections. The study of the mechanisms, consequences and possible therapeutic targets of long-term viral infections is fundamental, given the large number of chronic diseases associated with viral persistence.

The role of macrophages in hRSV infection is complex and depends on the cell phenotype, time post infection and interaction with other cells. Macrophage polarization is influenced by different mechanisms that include cytokine microenvironment and lipid metabolism. Recently, M1 polarization has been characterized by a high rate of glycolysis, whereas oxidative phosphorylation and fatty acid oxidation are characteristic of M2-like macrophages (Batista-Gonzalez et al., 2020). Viruses often evolve to promote M2-like responses in order to survive in host cells (Sang et al., 2015). In coculture experiments with human bronchial epithelial cells and blood derived macrophages, M2-like macrophages were shown to increase hRSV infection, while M1-like macrophages protected against epithelial infection (Ronaghan et al., 2022).

We previously reported the ability of hRSV to persist for over 150 passages in the P388D1 mouse macrophage-like cell line (Sarmiento et al., 2002; Ruiz-Gómez et al., 2021) and showed that hRSV-persistently infected macrophages (piMϕ) have a predominantly proinflammatory profile or M1-like status. Indeed, conditioned medium from piMϕ can induce a proinflammatory/antiviral state in non-infected macrophages due to the presence of biologically active inflammatory cytokines and chemokines (Rivera-Toledo et al., 2017). Nevertheless, arginase 1 (*Arg-1*), a marker of M2 polarization in macrophages, is highly expressed and shows increased enzymatic activity in piMϕ, which interferes with the production of nitric oxide (NO), a potent antiviral effector molecule (Santiago-Olivares et al., 2019). This suggests that this M2 phenotype marker could contribute to viral persistence. It is not known whether hRSV persistence can induce the expression of other M2-associated genes.

Calreticulin (CRT) is a multifunctional and ubiquitous chaperone with canonical functions in the endoplasmic reticulum that include Ca++ homeostasis and correct glycoprotein folding (Gelebart et al., 2005; Michalak et al., 2009). However, CRT has also been reported outside the endoplasmic reticulum, in the cytoplasm, cell surface and extracellular space (Gold et al., 2010). In addition to its housekeeping functions, CRT has gained much attention due to its strong immunomodulatory activities that range from anti-inflammatory to pro-inflammatory effects. CRT has been identified in a wide range of parasites and influences cell responses and host-pathogen interactions. Mammalian CRT and its fragments have been shown to be potent stimulators of macrophages, stimulating the production of proinflammatory cytokines (Duo et al., 2014; Hong et al., 2010; Esperante et al., 2023; Huang et al., 2013). Many of the immunomodulatory functions of mammalian CRT are mirrored by parasite CRT.

Helminths are parasites that have coevolved with their human host for millennia. This long-term interaction has resulted in helminth survival strategies that regulate the host immune response through immunomodulatory molecules (Gazzinelli-Guimaraes and Nutman, 2018). A recent review by Ryan et al. (2020) suggests that helminth-derived product represent a “untapped pharmacopeia.” CRT from several parasites has been studied in different disease scenarios such as cancer, immune evasion, immune response, vaccination, and immunomodulation (Esperante et al., 2023; López et al., 2010; Rzepecka et al., 2009; Winter et al., 2005). Evidence suggests that CRT homologues exhibit comparable functions across different species. For example, CRT from different parasites can inhibit the complement system, thus facilitating their survival within the host (Esperante et al., 2023; Ramírez-Toloza and Ferreira, 2017). Furthermore, both the protozoan parasite *Trypanosoma cruzi* CRT (TcCRT) and the helminth *Taenia solium* CRT (TsCRT) recombinant forms exhibit antitumoral properties (López et al., 2010; Schcolnik-Cabrera et al., 2020).

However, TcCRT is more efficient in inhibiting the complement system and has a stronger antitumoral effect in comparison to human CRT (López et al., 2010; Ramírez-Toloza et al., 2020). The difference in efficiency can be attributed in part to conformational rearrangements based in species-specific structural features (Peña Álvarez et al., 2020).

We previously cloned and expressed CRT from the helminth *Taenia solium* as a recombinant protein (rTsCRT) (Mendlovic et al., 2004). Given the potential of mammalian CRT to activate macrophages, we hypothesized that rTsCRT can trigger a proinflammatory immune response in piMϕ, potentially leading to antiviral activity. In this study we analyzed the transcriptome profile of piMϕ with special emphasis on the antiviral and inflammatory responses, as well as on arginine and lipid metabolisms. We examined different time points after initiation of macrophage culture to identify early and late onset genes. In addition, we investigated the effects of rTsCRT treatment on the viral load and transcriptional signature induced by persistent hRSV infection.

## 2 Materials and methods

### 2.1 Cell culture

The mouse macrophage-like P388D1 cell line obtained from the American Type Culture Collection (TIB-63, ATCC, Manassas, VA, United States) was acutely infected with the hRSV strain Long (VR-26, ATCC, Manassas, VA, United States) at a multiplicity of infection of 1 (M.O.I. 1). Surviving cells were cultured to establish the piMϕ, as previously described (Sarmiento et al., 2002). The piMϕ were maintained in RPMI-1640 (Gibco, Thermo Fisher Scientific, Waltham, MA, United States) supplemented with 5% fetal bovine serum (Biowest, Bradenton, FL, United States), 1% penicillin-streptomycin (Invitrogen, Thermo Fisher Scientific, Waltham, MA, United States) and 1 μM 2-mercaptoethanol (Sigma-Aldrich, Saint-Louis, MO, United States), at 37°C and 5% CO_2_. hRSV genome persistence has been continuously evaluated by conventional and quantitative RT-PCR (RT-qPCR) to determine expression of viral nucleoprotein (N) mRNA and through direct immunofluorescence to detect expression of nucleocapsid (N) and fusion (F) proteins (Ruiz-Gómez et al., 2021). The original P388D1 non-infected macrophages (niMϕ) were grown under similar conditions as piMϕ and used as the control group. This study was performed with piMϕ from passages 161–169 and niMϕ from passages 97–103.

### 2.2 Recombinant *Taenia solium* calreticulin

The full-coding region of the mature rTsCRT without the signal peptide was cloned, expressed and the resulting protein was purified as previously described with some modifications (Fonseca-Coronado et al., 2011). Bacteria (BL21) expressing rTsCRT were sonicated (50 W) 3 times in 20 mM Tris-HCl buffer pH 7.3, in presence of protease inhibitors (Complete, Roche, Indianapolis, IN, United States), DNase (30 μg) (Roche, Indianapolis, IN, United States) and RNase (1,300 U) (Sigma-Aldrich, Saint-Louis, MO, United States), and centrifugated at 13,000 × g for 10 min at 4°C. The recombinant protein (53 kDa) was purified by separation in 1.5 mm thick 10% gels (Tris-glycine) SDS-PAGE, followed by ZnSO_4_ 0.2 N-Imidazole 0.2 M + SDS 0.1% staining (Sigma-Aldrich, Saint-Louis, MO, United States). The enriched recombinant protein was excised from the gel and eluted at 5 mV/tube during 8 h using an electro-elutor model and electro-dialysed (422 Bio-Rad, Hercules, CA, United States). Endotoxins were measured using the Pierce Endotoxin Kit (Thermo Fisher Scientific, Waltham, MA, United States) following the manufacturer’s instructions. Protein quality was examined by SDS-PAGE and protein concentration was determined by the Lowry method. Purified rTsCRT was filtered using a 0.22 μM filter and kept at −70°C until use.

### 2.3 MTT assay

piMϕ and niMϕ were seeded in 96-well plates (Corning, Corning, NY, United States) (2 × 10^4^/well) and incubated overnight. Cells were rinsed with PBS and 0.22 μm-filtered rTsCRT was added at concentrations of 1, 2, 5 and 10 μg/mL in 200 μL of supplemented RPMI-1640. rTsCRT was maintained for 24 and 48 h and 20 μL of 3-[4,5-dimethylthiazole-2-yl]-2,5-diphenyltetrazolium bromide (MTT) solution (Biological Industries, Sartorius, Kibbutz Beit-Haemek, Israel) (5 mg/mL in PBS) were added. Following a 3 h incubation at 37°C, supernatants were discarded, and formazan crystals dissolved in 100 μL of DMSO (Merck, Rahway, NJ, United States) for 15 min. Absorbance was measured at 570 nm in a microplate reader (Biorad, Hercules, CA, United States). Percentage of cell metabolic activity was estimated by normalizing the optical density (OD) value of rTsCRT treated cells to the OD value of untreated cells ×100.

### 2.4 rTsCRT treatment

niMϕ and piMϕ were seeded in 12-well plates (Corning, Corning, NY, United States) (0.5 × 10^6^/well) and allowed to adhere overnight. The following day, cells were rinsed once with PBS and rTsCRT was added at a final concentration of 5 μg/mL in supplemented RPMI-1640. After 6, 24 and 48 h, supernatants were collected, centrifuged at 250 g for 5 min and transferred to a fresh tube. All supernatants were frozen at −80°C until use. Additionally, monolayers were treated with 500 μL of Trizol reagent (Invitrogen, Thermo Fisher Scientific, Waltham, MA, United States) for RNA extraction.

### 2.5 RNA extraction and gene expression analysis by RT-qPCR

Total RNA was obtained with the Trizol reagent according to the manufacturer’s instructions. Total RNA from non-treated piMϕ and niMϕ and treated with 5 μg/mL of rTsCRT for 6, 24 and 48 h were isolated. RNA concentration for each condition was adjusted to 100 ng/μL in RNase-free water. RNA quality was determined by the capillary electrophoresis system Agilent 2100 Bioanalyzer (Agilent Technologies, Santa Clara, CA, United States). Samples showed RIN values between 8.0–10.0 and were processed in triplicates in the Microarray Core Facility at the National Institute of Genomic Medicine (INMEGEN) to produce cDNA. The resulting cDNA was hybridized to GeneChip mouse Clariom S microarrays (Thermo Fisher, Waltham, MA, United States), which analyze gene-level expression of >20,000 well-annotated mouse genes.

Gene expression was evaluated by one step quantitative RT-qPCR using the Luna Universal Probe One-Step RT-qPCR Kit (NEB, M3005S) (New England Biolabs, Ipswich, MA, United States). Taqman gene expression assays were performed using commercially available primers and probes (Applied Biosystems, Austin, TX, United States) for Il10 (Mm00439614_m1), Il1b (Mm01336189_m1), Il6 (Mm00439653_m1), Tnf-α (Mm00443258_m1) Irg-1 (Mm01224532_m1), Il18 (Mm00434226_m1), Lcn2 (Mm01324470_m1), Cxcl2 (Mm00436450_m1), Socs3 (Mm00545913_s1) and CD40 (Mm00441891m1). Reactions consisted of a volume of 10 μL containing 2 μL RNA, 5 μL Luna Universal One-Step Reaction Mix, 0.5 μL Luna WarmStartRT Enzyme Mix, 0.5 μL each, forward and reverse primers and 1.5 μL RNase-free H_2_O; a LightCycler 2.0 (Roche, Indianapolis, IN, United States) was used. The parameters for PCR amplification were 95 °C for 10 min, followed by 45 cycles each consisting of denaturation at 95°C for 10 s, annealing at 60°C for 10 s and extension at 72°C for 10 s. The housekeeping gene Eef2 (Mm 01171435_gH) was used to normalize mRNA expression. Relative RNA quantitation was calculated using the ΔΔCt method (Livak and Schmittgen, 2001).

### 2.6 Microarray analysis

Microarray analysis was performed with the Transcriptome Analysis Console 4.0 (TAC) (Affymetrix, Thermo Fisher Scientific, Waltham, MA, United States). Genes with a fold change threshold of ±2 or higher and *p*-value ≤0.05 were considered for subsequent analyzes. DAVID (Huang et al., 2009) and Enrichr/Enrichr-KG (Xie et al., 2021; Evangelista et al., 2023) analyzes were performed with default settings (DAVID: Select_Identifier = Official_gene_symbol, Classification Stringency = Medium, Enrichment score threshold (EASE) = 1.0 for Functional Annotation clustering and 0.1 for Gene Ontology and Pathway predictions; Enrichr/Enrichr-KG: Top 30, 20 or 10 GO Biological Process 2021, Minimum libraries and links per gene = 1, Minimum links per term = 1, Subgraph size limit = 100). We obtained a list of the top affected biological processes by DAVID and Enrichr-KG, as well as a visualization of connections of enriched genes and processes with Enrichr-KG. All of them were selected according to their *p*-value (*p* ≤ 0.05) and previous association with inflammation, antiviral response, or metabolism.

### 2.7 Virus genome copies

Absolute quantification of the hRSV genome was performed with a plasmid DNA standard curve that includes an 85 bp region from the viral N gene (hRSV A strain Long, GeneBank accession AY911262.1). Specific primers and probe (FAM/BHQ1) (OligoT4, Irapuato, GT, Mexico) were designed to target such genomic region: forward, 5′-AATTTCCTCACTTTTCCAGTGTAG-3′; reverse, 5′-TGATTCCTCGGTGTACCTCTG-3′; probe, 5′GCAATGCTGCTGGCCTAGGCATAAT G-3′. One-step RT-qPCR reactions were carried out with SuperScript III Platinum One-Step RT-qPCR Kit (Invitrogen, Thermo Fisher, Waltham, MA, United States). A standard curve was constructed with 10-fold serial dilutions from a plasmid DNA stock with 1 × 10^7^ N gene copies (5 μL per reaction). Virus genome copies in piMϕ treated and untreated with rTsCRT were evaluated in triplicate reactions with 25 ng of total RNA, in a thermocycler StepOne Real-Time PCR System (Applied Biosystems, Austin, TX, United States). Cycling program was 50°C for 15 min, followed by 40 cycles of 95°C for 15 s and 60°C for 30 s. Mean Ct values were plotted versus the log10 of standard concentrations and unknown hRSV genome copies were determined by interpolation from the standard curve (equation: $y=-1.297\ln\left(x\right)+42.265;R^{2}=0.98$ ).

### 2.8 Cytokine assay

niMϕ and piMϕ growth media were collected at 6, 24 and 48 h of incubation with or without treatment with rTsCRT and stored at −80°C until use. Macrophage cultures were performed in triplicate. The concentration of TNF-α, IL-6, INF-γ, MCP-1, IL-10, and IL-12 was tested by the Cytometric Bead Array Mouse inflammation kit BDTM (BD Biosciences, San Jose, CA, United States) according to the manufacturer’s instructions.

### 2.9 Statistical analysis

Statistical analysis and graphs were performed with GraphPad Prism 9 (GraphPad Software Inc., San Diego, CA, United States). For parametric data, we used a one-way ANOVA to determine significant differences between groups. For comparison between 2 groups, a Student *t*-test for parametric and Mann–Whitney test for non-parametric data were used. A *p* ≤ 0.05 value was considered as a statistically significant threshold in all tests.

## 3 Results

### 3.1 Modified genes during hRSV persistence in murine macrophages

**Figure 1:**
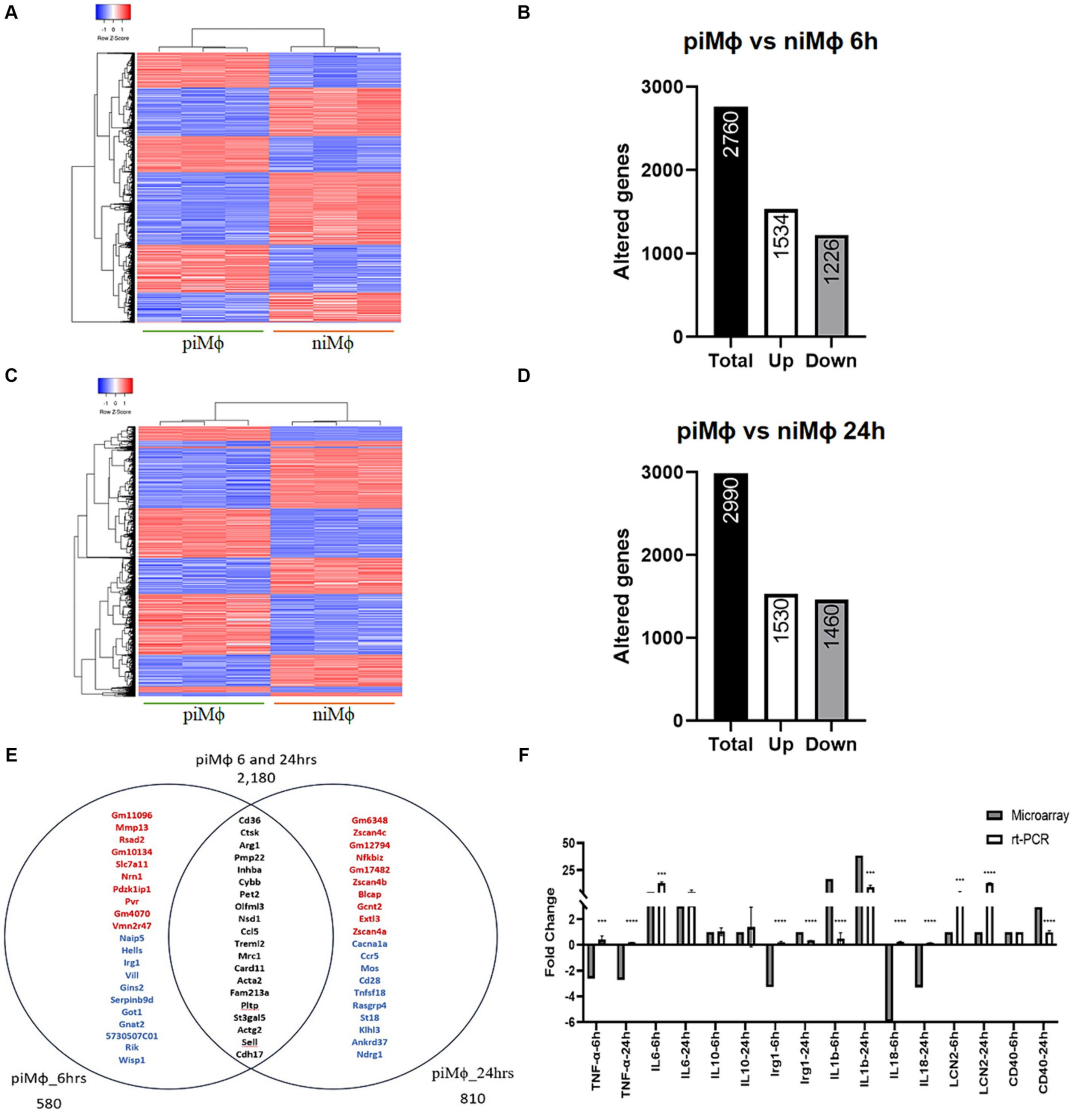
Time-dependent gene expression in persistent infection of hRSV versus niMϕ. **(A)** Heatmap representation of differentially expressed genes in piMϕ versus niMϕ after 6 h of *in vitro* culture. **(B)** Number of altered genes in piMϕ at 6 h of *in vitro* culture. **(C)** Heatmap representation of differentially expressed genes in piMϕ versus niMϕ after 24 h of *in vitro* culture. **(D)** Number of altered genes in piMϕ at 24 h of *in vitro* culture. **(E)** Venn diagram of genes expressed only at 6 or 24 h and at both timepoints. Top-up-regulated genes are shown in red, while top-down-regulated genes are shown in blue. Common genes expressed at both timepoints are shown in black. **(F)** Microarray expression data validation by qRT-PCR at 6 and 24 h. piMϕ , persistently infected macrophages with hRSV; niMϕ, non-infected macrophages; hRSV, human respiratory syncytial virus. *p*-values were calculated using the Student *t*-test between microarrays and rt-PCR data for each cytokine: *** = ≤0.001 and **** = ≤0.0001.

The gene expression profile was evaluated in niMϕ and piMϕ at 6 and 24 h. For the microarray analysis we first considered the effect of hRSV persistent infection, contrasting differentially expressed genes in piMϕ versus niMϕ. The heatmaps in Figure 1 show that piMϕ and niMϕ are clearly separated by the differentially regulated genes (Supplementary material S1). Virus persistence altered 2,760 genes in piMϕ cultured for 6 h of which 1,534 were upregulated and 1,226 were downregulated (Figures 1A,B). At 24 h, 2,990 genes were differentially expressed, 1,530 were upregulated and 1,460 were downregulated (Figures 1C,D). A total of 2,180 shared genes were identified at 6 and 24 h, while genes expressed only at 6 h or 24 h were 580 and 810, respectively (Figure 1E). These results are supported by RT-qPCR experiments where we observed a similar trend in the relative expression of several differentially expressed genes (Figure 1F).

**Figure 2:**
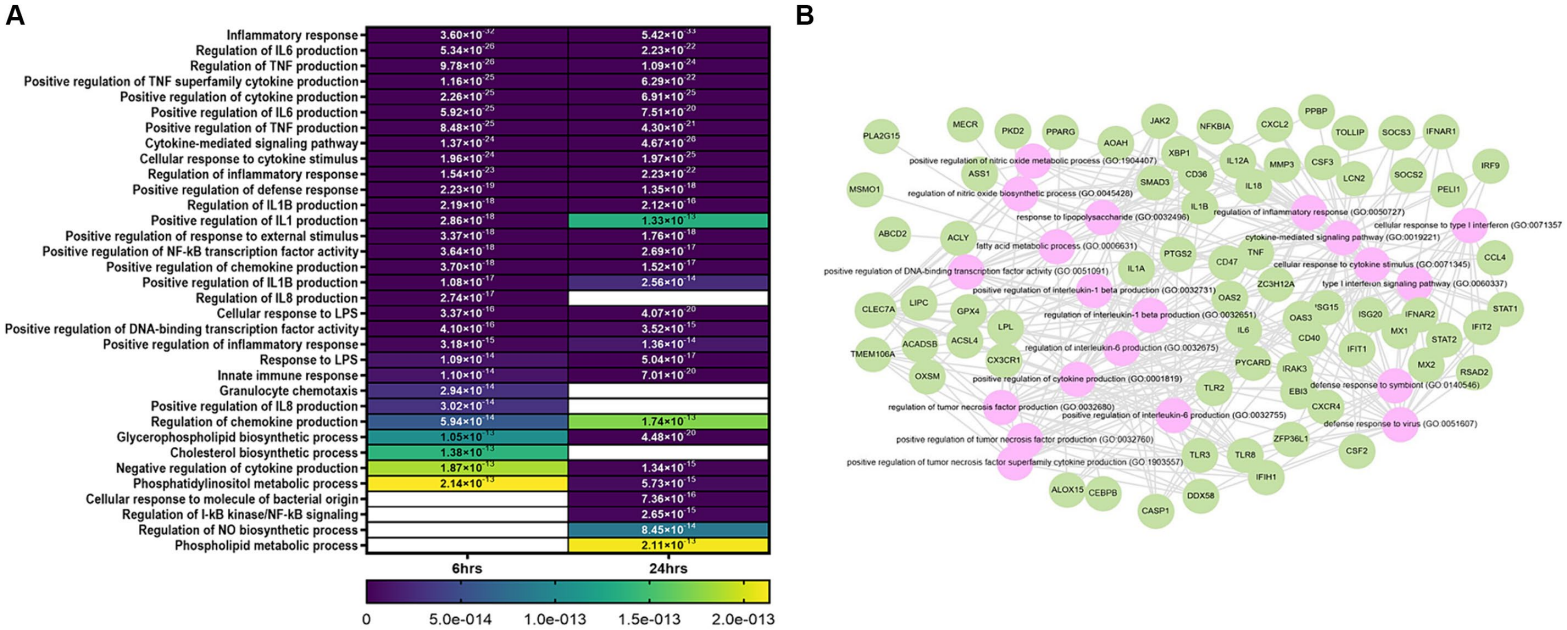
Bioinformatic analysis of niMϕ and piMϕ. **(A)** Heatmap of top biological processes altered after the infection with hRSV at 6 and 24 h. **(B)** Predicted network of relevant biological processes deregulated at both timepoints. Genes deregulated by hRSV infection (green) and predicted altered processes (pink) using DAVID and Enrichr/Enrichr-KG software. Blank cells represent the absence of the corresponding process at the specified time. hRSV, human respiratory syncytial virus. The values plotted in the heatmap correspond to the *p*-values of each process.

Functional annotation analysis showed that the main processes altered at 6 and 24 h were related to the inflammatory response, regulation of cytokines and chemokines, defense response to virus, positive regulation of transcription factor activity, and biosynthesis of products such as NO, cholesterol and glycerophospholipids (Figures 2A,B). Other modified processes were cell cycle, proliferation, and apoptosis (Supplementary material S2).

Defense response or antiviral genes of interest exclusively overexpressed at 6 h were *Rsad2*, *Oasl2*, *Isg15* and *Ifih1/Mda5*, whereas IFN-α and IFN-β receptor subunit 1 (*Ifnar1*), as well as genes that mediate inhibition of the inflammatory response, like *Irg1* and *Il10rb* were downregulated (Supplementary material S1). The Il1 receptor antagonist (*Il1rn*) mRNA was only induced at 6 h, suggesting this gene might have an early role in modulating the inflammatory response in piMϕ. Interesting genes exclusively overexpressed at 24 h were *Il12a*, *Cebpb* and CD40.

Common downregulated genes at 6 and 24 h (Supplementary material S1) were also associated with the antiviral response and immunomodulation in piMϕ, like *Ifnar2*, *Ddx58/Rig1*, *Oas2*, *Oas3*, *Mx1*, *Mx2*, *Isg20*, *Ifitm6*, *Parp9* and *Ebi3*. Despite the upregulation of the IFN-stimulated genes *Ifit2* and *Rnase2a*, they were concurrently expressed with the Jak/STAT pathway inhibitor, suppressor of cytokine signaling 2 (*SOCS2*). On the other hand, genes encoding RNA editing enzymes or their subunits such as *Apobec1*, *A1CF* and *Adat1* were downregulated at both time points, whereas the gene encoding the adenosine deaminase acting on RNA (*Adar*) was upregulated only at 24 h.

An additional group of genes steadily upregulated at 6 and 24 h were those involved in inflammatory activity, such as, *Cxcl2*, *Lcn2*, *Ccl4*, *Ccl6*, *Ccl12*, *Tlr3*, *Tlr2*, *Tlr8*, *Cxcr4*, *Tnfaip6 and Cd9*, while the negative modulator of inflammation *Ctla2a* increased up to 20.3-fold. Upregulated mRNA expressing cytokine genes at 6 and 24 h were *Il1a*, *Il1b* and *Il6*, while *Il18* and *Tnf-α* were downregulated. We also identified overexpressed genes involved in resistance to oxidative stress, like *Arg1*, *Oxr1* and *Nostrin* with up to 1784.2-, 6.9-and 2.8-fold-change, respectively.

Genes related to lipid metabolism were also highly modified in piMϕ. The highest upregulated gene was the scavenger receptor B CD36, with a fold-change value of 7,059 and 4,312 at 6 and 24 h, respectively. Other genes involved in lipid metabolism that were significantly upregulated included cathepsins (*Cts*), *Ctsk*, *Ctsh*, and *Ctsl* with fold changes of 2,757-2,171, 218-160, and 53-47, respectively. Lipocalin 2 (*Lcn2*) and peroxisome proliferator activated receptor-γ (*Pparγ*) (58–218 and 29–93, respectively) were also induced.

### 3.2 rTsCRT is non-cytotoxic for piMϕ versus niMϕ

We assessed whether rTsCRT causes cytotoxicity in niMϕ and piMϕ treated with concentrations of 1, 2, 5, and 10 μg/mL for 24 and 48 h. The metabolic activity was evaluated by the MTT assay. Alterations in cellular metabolism may change the ability of the NADPH-dependent cellular oxidoreductase enzymes to reduce MTT to formazan. Consequently, the OD measured in this assay indirectly reflects cellular metabolic activity, and a decrease in OD may be indicative of cytotoxicity. Results showed that 1, 2, and 5 μg/mL of rTsCRT did not reduce the metabolic activity in niMϕ and piMϕ at 24 or 48 h. In fact, both cell lines significantly increased metabolic activity (up to 27%) after treatment with 1 μg/mL after 48 h. Only 10 μg/mL of rTsCRT caused a modest, non-significant cytotoxic effect (~5%). Therefore, 1–5 μg/mL are suitable non-cytotoxic concentrations to stimulate piMϕ and niMϕ (Supplementary Figure S1).

### 3.3 Modified genes upon rTsCRT treatment

**Figure 3:**
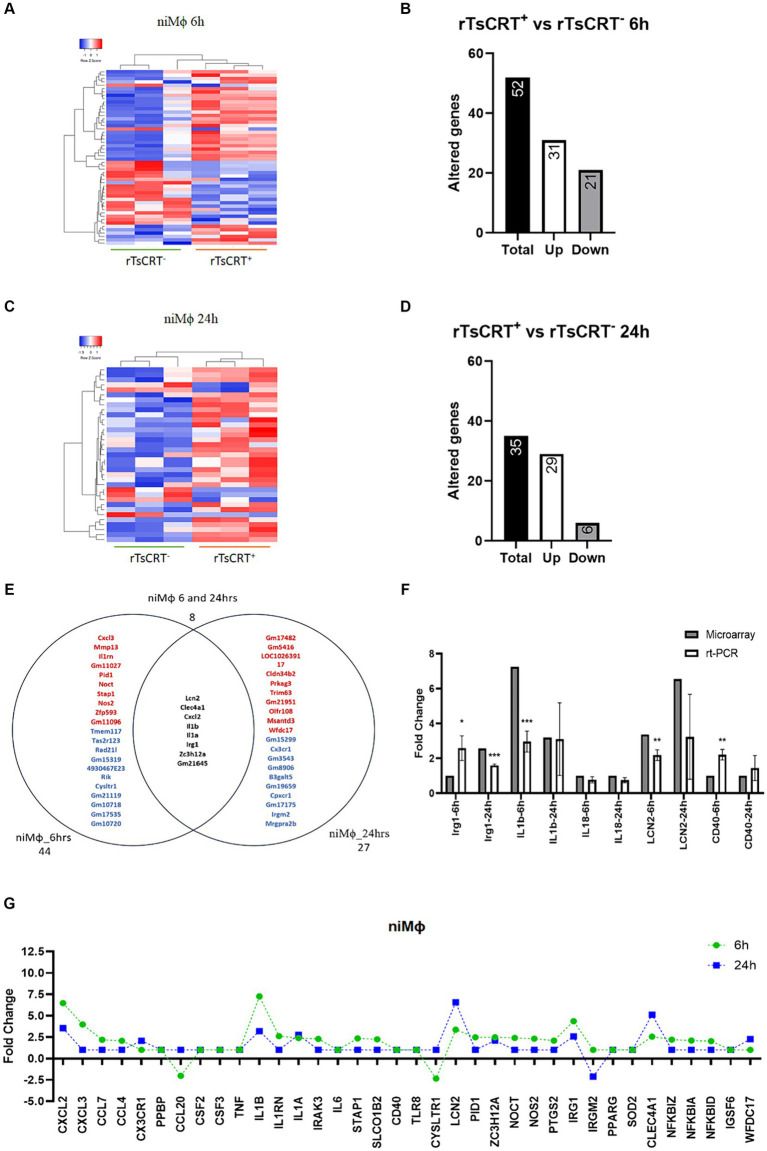
Time-dependent gene expression in niMϕ treated with 5 μg of recombinant *T. solium* calreticulin (rTsCRT^+^). **(A)** Heatmap representation of differentially expressed genes in niMϕ after rTsCRT treatment for 6 h. **(B)** Number of altered genes in niMϕ after rTsCRT treatment for 6 h. **(C)** Heatmap representation of differentially expressed genes in niMϕ after rTsCRT treatment for 24 h. **(D)** Number of altered genes in niMϕ after rTsCRT treatment for 24 h. **(E)** Venn diagram of the genes expressed only at 6 or 24 h and at both timepoints. Top-up-regulated genes are shown in red, while top-down-regulated genes are shown in blue. Common genes expressed at both timepoints are shown in black. **(F)** Microarray expression data validation by qRT-PCR at 6 and 24 h. **(G)** Temporary gene expression of niMϕ treated with rTsCRT. niMϕ , non-infected macrophages. *p*-values were calculated using the Student *t*-test between microarrays and rt-PCR data for each cytokine: * = <0.05, ** = <0.01, and *** = ≤0.001.

The effect of 5 μg/mL of rTsCRT on gene transcription in niMϕ and piMϕ was analyzed after 6 and 24 h. Differentially expressed genes in each condition were determined by comparison with their respective untreated counterparts. niMϕ showed 52 altered genes after 6 h of treatment of which 31 were upregulated (59.6%), while at 24 h the altered genes were 35, including 29 upregulated genes (82.8%) (Figures 3A–D; Supplementary material S3). Eight genes (10.1%) were shared between 6 and 24 h belonging to immunoregulatory and proinflammatory responses (Figure 3E). We validated the microarray expression data by RT-qPCR of some modified immune response genes (Figure 3F). Interestingly, rTsCRT induced a predominantly transitory inflammatory state in niMϕ, as genes like *Cxcl2*, *Il1b*, and *Irg1*, that were upregulated at 6 h reduced their expression by 42–57% at 24 h. *Cxcl3*, *Ccl4*, *Nos2*, *Irak3*, *Nfkbia*, *Nfkbid* and *Nfkbiz*, showed a 2–4-fold increase at 6 h and returned to basal levels at 24 h. Only the inflammatory genes *Il1a* and *Lcn2*, as well as the immunoregulatory gene *Clec4a* were expressed at higher levels at 24 h (Figure 3G).

**Figure 4:**
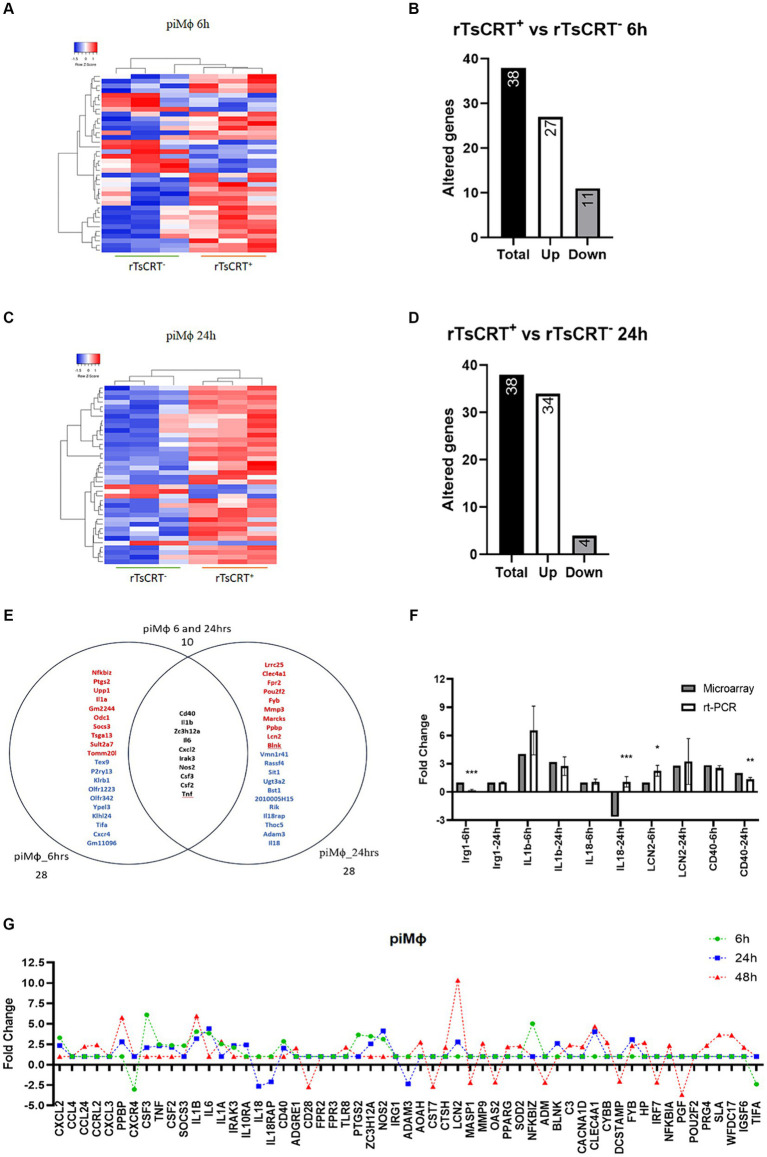
Time-dependent gene expression in piMϕ treated with 5 μg of recombinant *T. solium* calreticulin (rTsCRT^+^). **(A)** Heatmap representation of differentially expressed genes in piMϕ after rTsCRT treatment for 6 h. **(B)** Number of altered genes in piMϕ after rTsCRT treatment for 6 h. **(C)** Heatmap representation of differentially expressed genes in piMϕ after rTsCRT treatment for 24 h. **(D)** Number of altered genes in piMϕ after rTsCRT treatment for 24 h. **(E)** Venn diagram of the genes expressed only at 6 or 24 h and at both timepoints. Top up-regulated genes are shown in red, while top down-regulated genes are in shown blue. Common genes expressed at both times are in black. **(F)** Microarray expression data validation by qRT-PCR at 6 and 24 h **(G)** Temporary gene expression of piMϕ treated with rTsCRT. piMϕ , persistently infected macrophages with hRSV; hRSV, human respiratory syncytial virus. *p*-values were calculated using the Student *t*-test between microarrays and rt-PCR data from each cytokine: * = <0.05, ** = <0.01, and *** = ≤0.001.

In piMϕ we identified 38 altered genes at 6 and 24 h of treatment with rTsCRT, including 27 and 34 upregulated genes, respectively (Figures 4A–D; Supplementary material S4). Ten genes were shared (17.3%) between 6 and 24 h including *Zc3h12a* and those associated with proinflammatory responses such as *Nos2*, *Il1b*, *Irak3*, *Csf2*, *Csf3*, *Il6*, *Tnf-α*, *Cxcl2* and *Cd40* (Figures 4E,F; Supplementary material S4). *Socs3*, the inhibitor of STAT3 was expressed only at 6 h, while inflammatory genes such as *Lcn2*, *Ppbp* and *Mmp3*, as well as the immunoregulatory *Clec4a* and *Il10ra* were only upregulated at 24 h. These observations suggest that rTsCRT contributed to maintain an inflammatory phenotype in piMϕ (Figure 4G; Supplementary materials S3, S4).

To evaluate this premise, we treated piMϕ with rTsCRT for 48 h to assess the maintenance of the proinflammatory state. We observed the downregulation of 26 genes (Supplementary Figure S2), of which *Il6*, *Tnf-α*, *Cxcl2*, *Cd40*, *Nos2*, *Zc3h12a*, *Irak3*, *Csf2* and *Csf3* “returned to basal levels, whereas *Il1b*, *Il1a*, *Ppbp*, and *Lcn2* displayed a 2–3.7-fold increase as compared to their expression at 24 h. Furthermore, rTsCRT induced expression of *Pparg*, *Igsf6*, *Tlr8*, *Wfdc17*, *Sod* and *Nfkbia* mRNA exclusively at 48 h (Figure 4G; Supplementary material S4).

**Figure 5:**
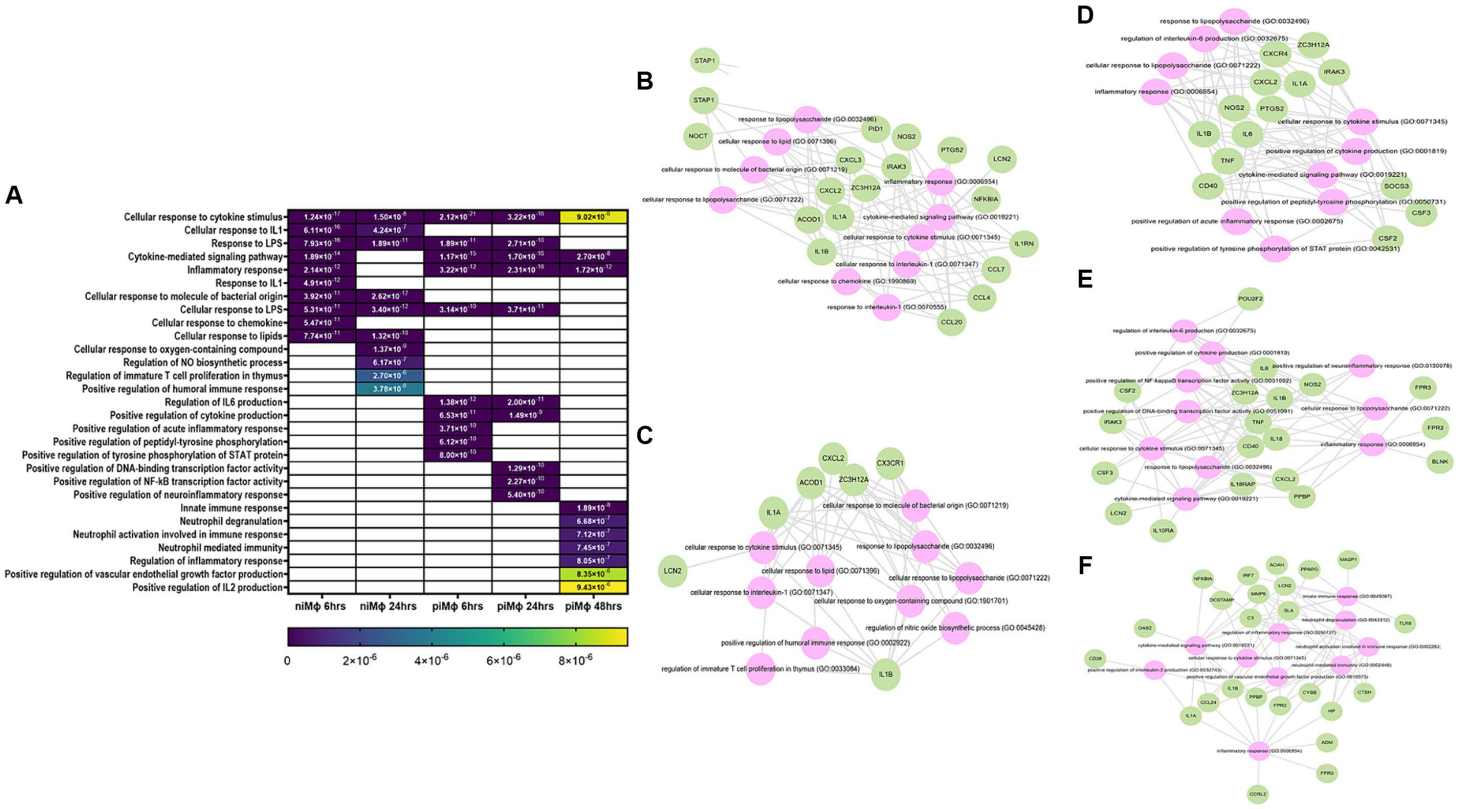
Bioinformatic analysis of rTsCRT-treated (rTsCRT^+^) versus non-treated (rTsCRT^−^) macrophages. **(A)** Heatmap of biological processes altered after the treatment of niM ϕ and piMϕ with rTsCRT at 6, 24, or 48 h. **(B,C)** Predicted network in niMϕ at 6 h and 24 h. **(D,E)** Predicted network in piMϕ at 6 h and 24 h. **(F)** Predicted network in piMϕ at 48 h. Genes deregulated by hRSV infection (green) and the predicted altered processes (pink) using DAVID and Enrichr/Enrichr-KG software. Blank cells represent the absence of the corresponding process at the specified timepont. hRSV, human respiratory syncytial virus; rTsCRT, recombinant *T. solium* calreticulin; niMϕ, niMϕ, non-infected macrophages; piMϕ, persistently infected macrophages. The values plotted in the heatmap correspond to the *p*-values of each process.

The main processes affected in niMϕ and piMϕ by treatment with rTsCRT were related to the inflammatory response, as well as the cellular response to cytokine stimulus (Figure 5A). Processes such as the cellular response to lipids, cellular response to oxygen-containing compounds and regulation of NO biosynthesis were only activated in niMϕ (Figures 5A–C; Supplementary materials S5, S6). In contrast, processes related to positive regulation of cytokine production, regulation of IL-6 production, positive regulation of transcription factors like STAT and NF-kB, regulation of neutrophil activity coupled to IL-2 production and the positive regulation of ERK1/ERK2 cascade were only induced in piMϕ (Figures 5A–F; Supplementary material S6).

### 3.4 rTsCRT treatment increases cytokine production and reduces viral load in hRSV in piMϕ

**Figure 6:**
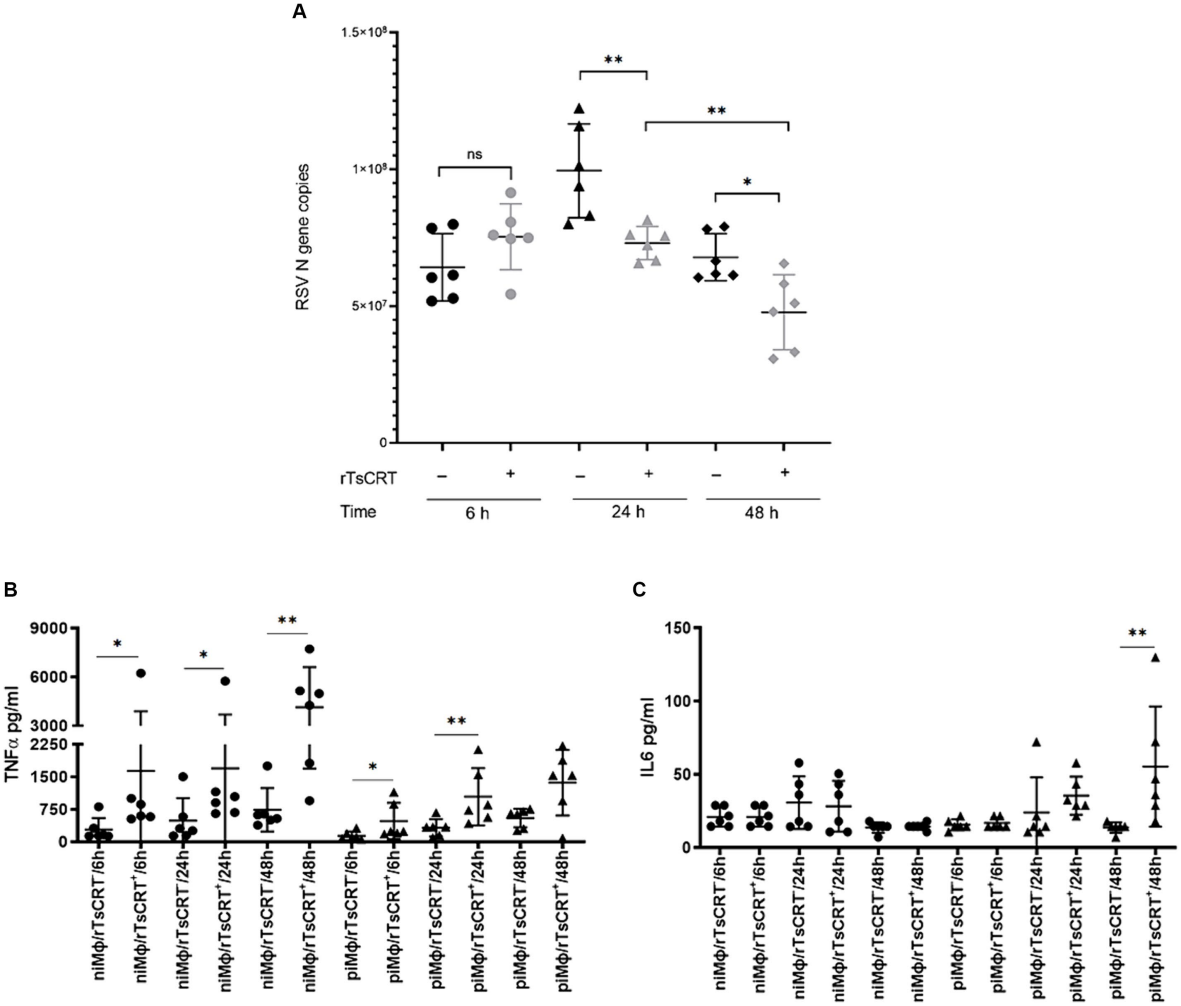
Viral replication and cytokine levels after rTsCRT treatment. **(A)** hRSV gene copies at 6, 24 and 48 h. **(B)** TNF-α and **(C)** IL-6 levels in niMϕ and piMϕ at 6, 24 and 48 h after culture. hRSV, human respiratory syncytial virus; rTsCRT, recombinant *T. solium* calreticulin; niMϕ, non-infected macrophages; piMϕ, persistently infected macrophages. *p*-values were calculated using the Student *t*-test between treated and untreated piMϕ for RSV gene copies and the Mann–Whitney test was used for cytokine concentrations between niMϕ and piMϕ, *p < 0.05 and **p < 0.01.

After observing that the inflammatory and immune responses are the main biological processes affected by rTsCRT, we evaluated the implication of the exacerbated proinflammatory environment induced by rTsCRT treatment on virus replication in piMϕ treated for 6, 24 and 48 h. Figure 6A shows that rTsCRT treatment significantly reduced the number of hRSV genome copies at 24 and 48 h in 36 and 42%, respectively, in comparison to non-treated piMϕ. Comparison between piMϕ treated for 24 h and 48 h displayed a significant progressive reduction in virus genome replication of 53%, suggesting that rTsCRT might control hRSV replication through activation/exacerbation of the inflammatory immune response. Accordingly, we observed a statistically significant higher production of TNF-α in niMϕ and piMϕ treated with rTsCRT for 6, 24 and 48 h as compared to non-treated controls (Figure 6B). Additionally higher levels of IL-6 were found in treated piMϕ at 48 h (Figure 6C). We found no differences in the levels of IFN-γ, MCP-1, IL-10 and IL12-p70 in piMϕ as compared to niMϕ (data not shown).

## 4 Discussion

In this study we investigated the transcriptional signature of piMϕ and found that persistent hRSV infection turns on predominantly genes involved in inflammation, antiviral response, as well as arginine and lipid metabolism, suggesting that piMϕ attain a mixed M1/M2 profile. Additionally, we analyzed the transcriptional changes induced by rTsCRT treatment and showed further stimulation of the inflammatory response. This stimulation was associated with a reduction in the number of hRSV copies in piMϕ.

In addition to epithelial cells, hRSV can infect both human and mouse alveolar macrophages (AMϕ) (de Souza et al., 2019; Johnson et al., 2007; Makris et al., 2016; Ravi et al., 2013). Evidence suggests that macrophage infection is temporarily productive as infective viruses are detected at very low titers (≤2 × 10^1^ PFU/mL). Subsequently, it changes to a nonproductive state (absence of virions) with persistent expression of viral proteins, while recovery of viral RNA is sustained (Sarmiento et al., 2002; Ravi et al., 2013). The low-to-nonproductive hRSV infection in macrophages might not be important to propagate the virus. Nevertheless, viral proteins and nucleic acids persist as chronic stimuli for the immune system (Rivera-Toledo et al., 2017; de Souza et al., 2019; Ravi et al., 2013).

During acute hRSV infection viral dsRNA is detected by RIG-I and MDA5 to activate IRF3, which translocates to the nucleus and promotes transcription of IFN-β. IFN-β interacts in both autocrine and paracrine manners through the heterodimeric receptor composed of the IFNAR1 and IFNAR2 subunits to activate the Jak/STAT signaling pathway. Ultimately, the heterotrimeric interferon-stimulated gene factor 3 (ISGF3), composed of STAT-1, STAT-2 and IRF9 is assembled and translocates to the nucleus to initiate the transcription of multiple interferon-stimulated genes (David et al., 1993). Interestingly, in this study we observed that piMϕ downregulated genes involved in the recognition of viral RNA and genes transcribed in response to IFN-I, such as *Ddx58/Rig1*, *Ifnar1*, *Ifnar2*, *Stat1*, *Stat2* and *Irf9*. Conversely, the expression levels of *Ifna* and *Ifnb* remained unaltered. Previously, we reported that IRF3 is active in piMϕ, as it is normally phosphorylated and located in the nucleus. However, piMϕ do not respond to either endogenous or exogenous IFN-β, as demonstrated by the lack of STAT-1 phosphorylation after a 45 min stimulation, thus limiting the expression of antiviral genes (Rivera-Toledo et al., 2015). A main function of the hRSV nonstructural proteins 1 and 2 (NS1 and NS1) is to evade the IFN-I-mediated antiviral response by targeting RIG-I, MAVS and STAT-2, as well as to induce expression of suppressor of cytokines signaling 1–3 (SOCS 1–3) that blocks the activation of the Jak/STAT pathway (Hashimoto et al., 2009; Sedeyn et al., 2019). It was recently described that NS1directly binds to regulatory elements of immune response genes during hRSV infection, suppressing their transcription (Pei et al., 2021). The antiviral transcriptomic profile reported herein agrees with these data. We observed either downregulation or transitory expression of many antiviral genes. However, genes such as *Ifitm10*, *Ifi203*, *Ifi205*, *Ifit1* and *Ifit2* showed an upregulated transcription at 6 and 24 h, suggesting that expression of some antiviral genes is regulated by alternative pathways to ensure host-cell survival during viral infections. Further underscoring the importance of antiviral pathways in controlling hRSV replication. The balance of an active immune system, capable of limiting virus replication, and the ability of the virus to dampen such antiviral responses are essential conditions to prevent cell death and facilitate long-term viral infections (Griffin, 2022).

In addition to IRF3, NF-kB can be activated by ligands of TLR3 in a MyD88-independent manner and by signaling via the IL-1/IL-1-Receptor through adaptor proteins expressed by *Myd88*, *Tollip* and *Peli1*. The latter two genes, along with *Tlr3* were upregulated in piMϕ. Hence, IRF3 and NF-kB may be key transcription factors controlling the long-term interaction between the virus and host cells. IRF3 does not exclusively target type-I interferon genes, it also induces the transcription of other antiviral genes, as well as the chemokine *Ccl5* that has been shown to have antiapoptotic properties in macrophages (Diamond and Farzan, 2013; Grandvaux et al., 2002; Jiang et al., 2004; Tyner et al., 2005). Accordingly, while we did not observe alterations in the expression of *Ifn α/β*, the ISGF3-related genes were downregulated in piMϕ. Overexpression of *Ccl5* and some antiviral genes could be explained by an ISGF3-independent signaling pathway, potentially involving direct activity of IRF3 and/or NF-kB (Diamond and Farzan, 2013). In fact, NF-kB activation is induced by diverse stimuli making it an essential transcription factor that mediates responses to physiological stress (Pahl, 1999). Promoters of many interferon-stimulated genes (ISGs) have binding sites for NF-κB and cooperate with IRFs to establish the antiviral state (Antonczyk et al., 2019).

Other target genes for NF-kB include cytokines, chemokines, effectors of the oxidative stress and apoptosis, immunoreceptors and transcription factors (Pahl, 1999; Nilsson-Payant et al., 2021). In piMϕ we identified upregulation of many proinflammatory cytokine and chemokine genes, like *Il1a*, *Il1b*, *Il6*, *Il12a*, *Ccl2*, *Ccl4*, *Ccl7*, *Ccl12*, *Cxcl2*, *Cxcl10* and *Cxcl14*. These proinflammatory cytokines are associated with a M1 phenotype in macrophages. In contrast, *Tnf-α*, *Il18* and *Il18* receptor accessory protein (*Il18rap*) that also have a central function as proinflammatory mediators, were downregulated (Rex et al., 2020). One pathway that can induce expression of TNF-α, IL-1α, IL-1β and IL-6 is through IL-18/IL-18 receptor and IL-18rap (Tsutsui et al., 1997). We found that the components of the IL-18 signaling pathway were downregulated in piMϕ. Accordingly, expression of Tnfa is negative regulated in piMϕ. However, we detected overexpression of *Il1a*, *Il1b* and *Il6*, suggesting that alternative pathways can compensate for the reduced activity of the IL-18 signaling in inducing these cytokines. Additionally, the apoptosis-associated speck-like protein containing a CARD domain (Asc or Pycard) and the caspase 1 genes, both related to processing of the pro-IL1β through the Nlrp3 inflammasome (Schroder and Tschopp, 2010) were downregulated, suggesting that Il1β might not be in its bioactive form. Indeed, we have observed very low levels of IL-1β in conditioned medium from piMϕ (Rivera-Toledo et al., 2017).

We compared 3 earlier transcriptional studies conducted in mouse macrophages and A549 epithelial cells acutely infected with hRSV for 20–24 h with our results in piMϕ (Supplementary Table S1). These studies were mainly focused on genes related to the immune response. We identified equivalent fold-change expression of *Il1a*, *Il1b*, *Il6*, and some antiviral genes in piMϕ and acute hRSV infection. Interestingly, we observed the downregulation of genes such as *Tnf-α*, *Il18*, *Mx1*, *Rig1*, *Pkr*, *Stat1*, *Stat2* and *Irf9*, which appear to be hallmarks of hRSV persistence in contrast to the acute infection.

During early stages of hRSV infection TNF-α plays a protective role in the control of viral replication, while its chronic production can cause severe illness through immunopathology (Neuzil et al., 1996; Rutigliano and Graham, 2004). Therefore, TNF-α has a critical role in development and outcome of the hRSV infection and its expression is tightly regulated. The transcriptional regulator BCL3 that belongs to the IkB protein family, modulates *Tnf-α* expression by recruiting histone deacetylases to its promoter and induce a repressive chromatin state (Walker et al., 2013). We identified upregulation of Bcl3 gene at 6 and 24 h, accompanied by overexpression of the histone deacetylases (Hdac)-2, 8 and 10 in piMϕ. These observations suggest that these genes may participate in the pathway responsible for downregulating *Tnf-α* transcription. However, these findings require experimental validation.

Concerning proteins related to the transcription factor NF-kB, we identified upregulation of *Nfkb1* (p50), *Nfkbia* (Ikba) and *Nfkbiz* (IκBζ). Previous studies have shown that inflammatory stimuli induce the expression of IκBζ in monocytes/macrophages (Sundaram et al., 2016). IκBζ can subsequently associate with p50 homodimers bound to the IL-6 promoter, facilitating its transcription (Kamata et al., 2010). These mechanisms may contribute to the upregulation of IL-6 mRNA in piMϕ. The role of IL-6 during viral infections may be protective, by inhibiting virus replication or deleterious, through a synergistic interaction with IL-17 that induces antiapoptotic molecules avoiding elimination of infected cells and promoting long-term infections (Velazquez-Salinas et al., 2019). Although IL-6 has been associated with hRSV severe disease in infants, its participation in virus persistence has not been studied (McNamara et al., 2004).

The transcriptional profile in piMϕ suggests the involvement of multiple mechanisms to maintain hRSV persistence, including inhibition of *Tnf-α* expression and the IFN-I response. The apparent paradoxical upregulation of some antiviral genes, such as the RNA editing enzyme *Adar1*, *Isg20*, and genes within the IFIT and IFITM families (*Ifit1*, *Ifit2*, and *Ifitm10*), which restrict viral entry by altering membrane fusion and virus genome replication, may indicate molecular strategies used by the virus to modulate its own replication and persistence. For instance, ADAR1 induces hypermutation (A-to-G substitutions) in the matrix gene of the measles virus, resulting in its defective expression. This leads to a lack of infectious viral particles and persistence of virus genome in the central nervous system, resulting in fatal subacute sclerosing panencephalitis (Samuel, 2012). These findings are consistent with our previous results, which show defective virus production and a lack of syncytia formation in piMϕ. Additionally, a high proportion of A-to-G transitions is a hallmark of the persistent hRSV genome (GenBank accession no. MT492011 and MT492012) (Sarmiento et al., 2002; Ruiz-Gómez et al., 2021).

Genes associated with arginine metabolism also showed profound alterations during hRSV persistence. Notably, *Arg1* ranked third among the genes with the highest transcription fold change, in agreement with our earlier findings (Santiago-Olivares et al., 2019). In the arginine biosynthetic pathway, nitric oxide synthase 2 (NOS2) produces nitric oxide (NO) and citrulline from L-arginine. Then, argininosuccinate synthase 1 (ASS1) recycles citrulline to produce argininosuccinate, which is the substrate of arginosuccinate lyase (Rath et al., 2014). An increased expression of the *Ass1* gene in piMϕ suggests that arginine metabolism is active to allow bioavailability of this amino acid as a NOS2 substrate, since NO is essential to inhibit viral infection. Nevertheless, overexpression of Arg1 that has higher affinity for arginine compared to that of NOS2, can importantly divert the synthesis of NO contributing to hRSV persistence (Santiago-Olivares et al., 2019).

Macrophages with an M2 phenotype are associated with expression of *Arg1* and synthesis of ornithine, proline and polyamines (Martí et al., 2021) that modulate inflammation and in tissue repair (Kieler et al., 2021). By overexpressing *Arg1*, hRSV can shift the balance of arginine metabolism to the production of polyamines that may participate in genome packaging and activity of viral enzymes (Tomé, 2021), to ensure persistence and concomitantly evade elimination by NO. Indeed, ornithine aminotransferase (OAT) and ornithine decarboxylase (ODC) that produce proline and glutamate and polyamines, respectively (Caldwell et al., 2015) are upregulated in piMϕ.

**Figure 7:**
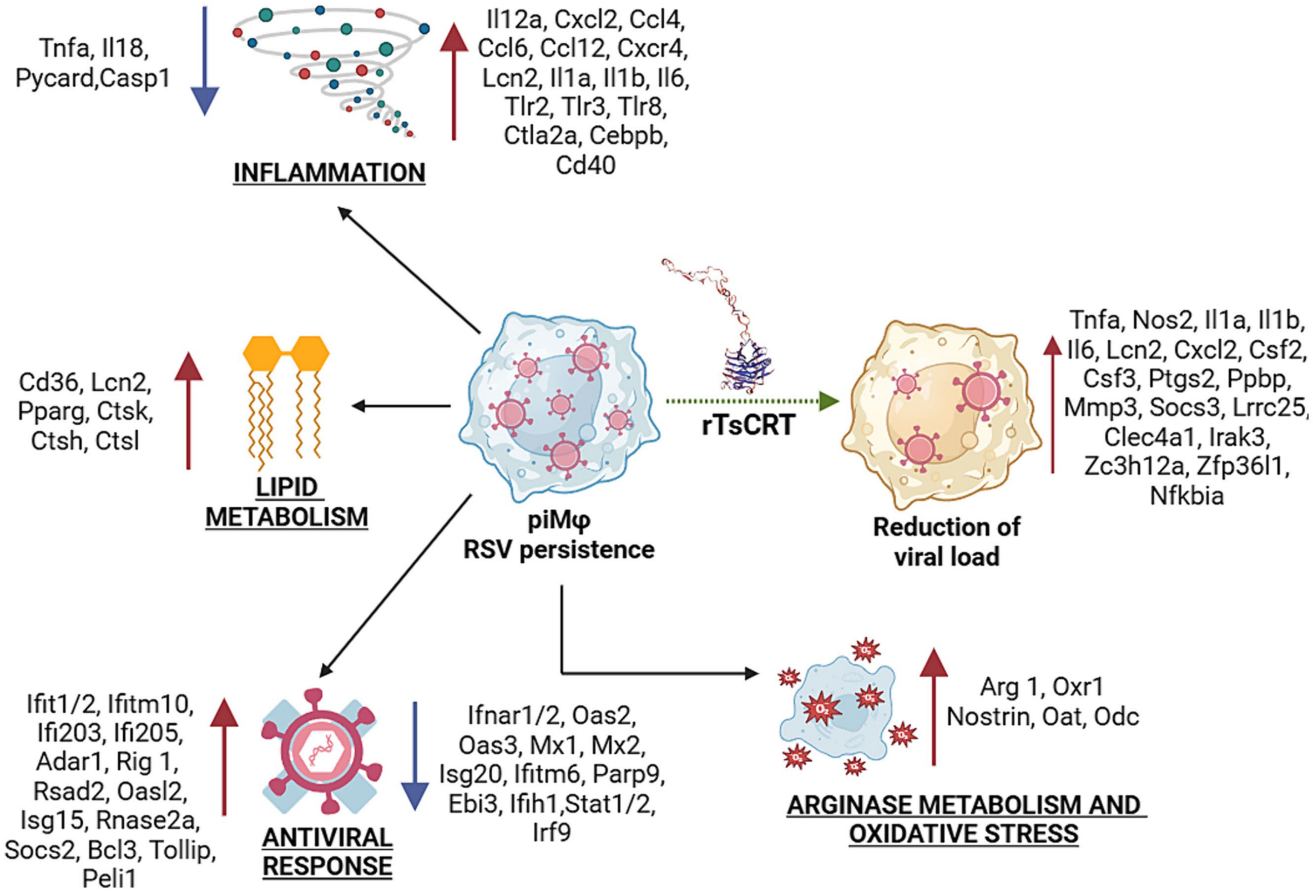
Transcriptional signature during hRSV persistence in macrophages and after treatment with rTsCRT. Inflammation, antiviral response, oxidative stress, lipid and arginase metabolisms are biological processes altered during virus persistence. Up- and down-regulation of proinflammatory, antiviral and tolerogenic or immunomodulatory genes suggest a gene expression balance to allow virus-host coexistence, particularly, promoting a mixed M1/M2 phenotype in piMϕ. Treatment with rTsCRT boosted the proinflammatory/immunomodulatory transcriptome, involving up-regulation of *Il1a*, *Il1b*, *Nos2* and *Tnf-α*, which might mediate the reduction of viral load. rTsCRT, recombinant *T. solium* calreticulin.

The anti-inflammatory M2 phenotype is also favored by fatty acid oxidation (FAO) and oxidative phosphorylation (Batista-Gonzalez et al., 2020). Our transcriptome analysis showed upregulated expression of neutrophil gelatinase–associated lipocalin 2 (*Lcn-2*), peroxisome proliferator activated receptor-γ (*Pparγ*), as well as CD36, all of which are involved in FAO (Chawla, 2010; Jin et al., 2011; Lim et al., 2006). Moreover, piMϕ upregulated the cysteine proteases cathepsins (*Cts*) K, L and B, shown to promote a M2 macrophage phenotype in tumor-associated macrophages (Li et al., 2019). M2 polarization is induced by several viruses as an immune evasion mechanism for efficient replication (Sang et al., 2015; Yu et al., 2022). In alveolar macrophages, hRSV can polarize the initial M1 response to an M2 phenotype that can promote persistent infection and continuous activation of the immune response (Wang et al., 2022). Thus, the antiviral and inflammatory responses together with modulation of the arginine and lipid metabolism pathways contribute to viral persistence and host cell survival (Figure 7).

rTsCRT potentiated the proinflammatory state induced by the presence of the replicating hRSV in piMϕ. *Ila*, *Il1b*, *Lcn2*, *Cxcl2* and *Nos2* expression were stimulated in both niMϕ and piMϕ, while *Il6*, *Tnf-α* and *Socs3* were upregulated only in piMϕ. The proinflammatory effects of rTsCRT treatment were accompanied by the expression of negative modulators such as *Socs3*, leucine rich repeat containing 25 (*Lrrc25*) in piMϕ, as well as *Zc3h12a*, a RNase that post-transcriptionally regulates *Il6* mRNA, along *Clec4a1*, and *IRAK3* in both niMϕ and piMϕ. Expression of zinc finger protein 36, C3H type-like 1 (*Zfp36l1*), critical for the decay of the mRNAs for TNF-α, was induced by rTsCRT treatment after 48 h (Matsushita et al., 2009). Thus, treatment with rTsCRT induced a proinflammatory phenotype accompanied by intrinsic regulation of the inflammatory response.

Treatment with rTsCRT resulted in a reduction in the viral load in piMϕ. This effect was accompanied by the induction of proinflammatory cytokine and chemokine genes, as well as by the production of TNF-α and IL-6 in rTsCRT-treated piMϕ. Accordingly, the transcriptomic analysis showed that rTsCRT reversed the inhibition of *Tnf-α* expression induced by viral persistence and upregulated the expression of *Tnf-α* in rTsCRT-treated piMϕ. TNF-α has been shown to have antiviral activity for several viruses including hRSV and can stimulate the NFκB and MAPK pathways, as well as genes such as *Il1b*, *Il6*, *Csf2*, *Ptgs2*, *Socs3* and *Nfkbia*, all of which were upregulated in rTsCRT-treated piMϕ (Figure 7). Indeed, mouse recombinant CRT has been shown to stimulate the production of TNF-α in mouse peritoneal macrophages by activating IκBα-NFκB and the c-Jun N-terminal kinase (JNK), as well as the production of IL-6 by phosphorylating the extracellular signal-regulated kinase (ERK) (Duo et al., 2014).

In addition to the upregulation of IL-6 and TNF-α, rTsCRT stimulated *Nos2* expression and we observed a marginally higher production of nitrites at 48 h after rTsCRT treatment (Supplementary Figure S3). Accordingly, mammalian recombinant CRT and its fragments can induce the accumulation of nitrites in peritoneal macrophages (Huang et al., 2013). NO has been shown to interfere with hRSV replication in both epithelial and macrophage cell lines (Santiago-Olivares et al., 2019; Kao et al., 2001). Thus, the effects on stimulation of cytokine and chemokine expression, as well as on *Nos2* expression, could explain the observed rTsCRT antiviral activity. The mechanism of action and the receptors involved in mediating the antiviral effects of rTsCRT need to be elucidated. Several receptors have been implicated in binding CRT, including low-density lipoprotein receptor (LRP-1), scavenger receptor A and F, and TLR-4 (Berwin et al., 2004; Berwin et al., 2003; Gardai et al., 2003; Li et al., 2015; Ogden et al., 2001). Further analysis is required to identify the receptors for rTsCRT and define the signaling pathways involved in its antiviral effect.

## 5 Conclusion

hRSV persistent infection altered the expression of many genes. The hRSV model of persistence in macrophages showed downregulation of many genes associated with the antiviral response. Paradoxically, we also observed overexpression of a few antiviral genes, RNA editing enzymes, as well as proinflammatory cytokines and chemokines that induce an M1 phenotype and can control viral replication. Concomitantly, lipid metabolism-related genes, together with *Arg1*, which collectively promote an M2 status were overexpressed. This suggests that hRSV persistence induces a mixed M1/M2 phenotype characterized by a weakened antiviral response. Viral persistence depends on mutual adaptation between the virus and the host-cell to succeed in long-term infection. The seemingly contradictory effects on the macrophage transcriptome may represent virus-induced mechanisms aimed at modulating its own replication while simultaneously evading the immune response. Thus, hRSV persistence appears to involve a balance between pro-inflammatory and anti-inflammatory signals to control viral replication and ensure the survival of both the infected cell and the virus. Remarkably, rTsCRT showed that piMϕ are not refractory to external stimuli and that the apparent dampening of immune response signaling pathways is still reversible, resulting in a significant reduction in viral genome copies. Our observations have implications for the development of targeted therapies aimed at eliminating or controlling persistent viral infections often associated with immunopathology.

## Data Availability

The original contributions presented in the study are included in the article/Supplementary material, further inquiries can be directed to the corresponding author.

## Supplementary material

SUPPLEMENTARY FIGURE S1 Cell viability after rTsCRT treatment. **(A,B)** niMϕ treated at 24 and 48 h respectively. **(C,D)** piMϕ treated at 24 and 48 h respectively. rTsCRT, recombinant *T. solium* calreticulin; niMϕ, niMϕ, non-infected macrophages; piMϕ, persistently infected macrophages with hRSV; hRSV, human respiratory syncytial virus. *p*-values were calculated using the ANOVA test: * = <0.05, **** = ≤0.0001.

SUPPLEMENTARY FIGURE S2 Time-dependent gene expression of macrophages with persistent infection of hRSV treated with 5 μg of recombinant calreticulin from *T. solium* (rTsCRT). **(A)** Heatmap of transcripts altered in piMf by rTsCRT treatment at 48 h. **(B)** Number of mRNAs deregulated in piMf treated with rTsCRT at 48 h. piMϕ, persistently infected macrophages with hRSV; hRSV, human respiratory syncytial virus.

SUPPLEMENTARY FIGURE S3 Production of nitrites in niMϕ and piMϕ after treatment with rTsCRT for 6, 24, and 48 h. rTsCRT, recombinant *T. solium* calreticulin; niMϕ, non-infected macrophages; piMϕ, persistently infected macrophages with hRSV; hRSV, human respiratory syncytial virus. *p*-values were calculated by the Mann-Whitney test between niMϕ or piMϕ treated with rTsCRT and their respective control group, none showed statistical significance.
