# Supplementary figures and images for: Transcriptome profiling of macrophages persistently infected with human respiratory syncytial virus and effect of recombinant Taenia solium calreticulin on immune-related genes
Source: Front Microbiol. 2024 Sep 4;15:1402589. doi: 10.3389/fmicb.2024.1402589 (PMC11408361; doi:10.3389/fmicb.2024.1402589)

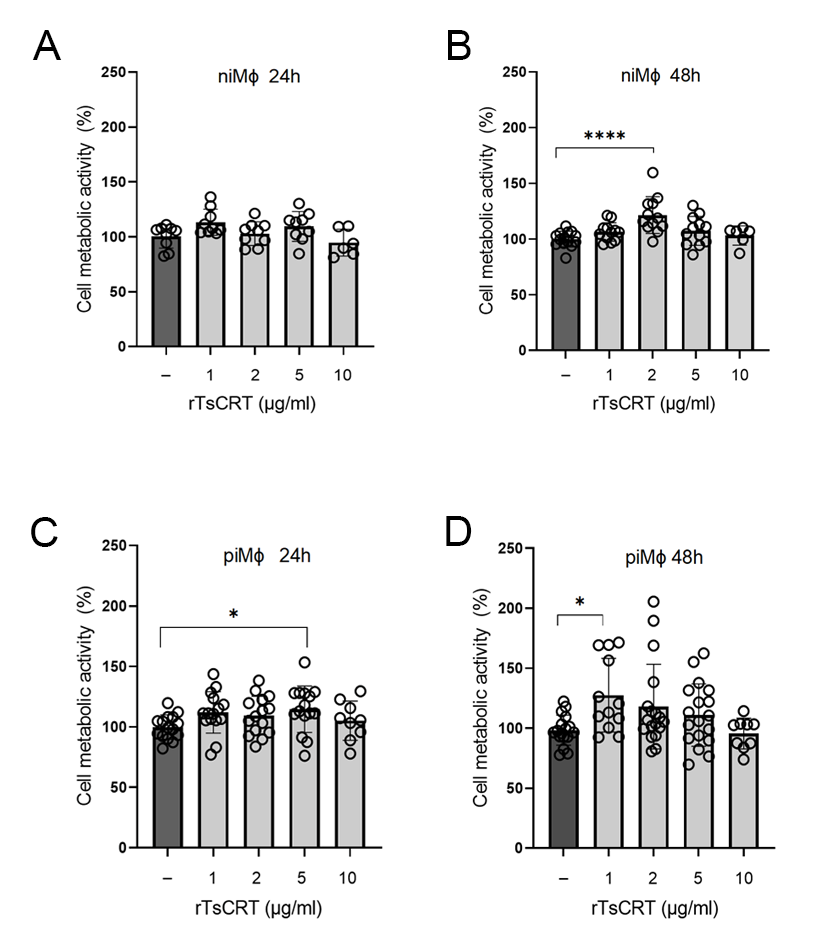

Supplement: SUPPLEMENTARY FIGURE S1 — Cell viability after rTsCRT treatment. (A,B) niMϕ treated at 24 and 48 h respectively. (C,D) piMϕ treated at 24 and 48 h respectively. rTsCRT, recombinant T. solium calreticulin; niMϕ, niMϕ, non-infected macrophages; piMϕ, persistently infected macrophages with hRSV; hRSV, human respiratory syncytial virus. p-values were calculated using the ANOVA test: * = <0.05, **** = ≤0.0001. [file Image_1.TIF]

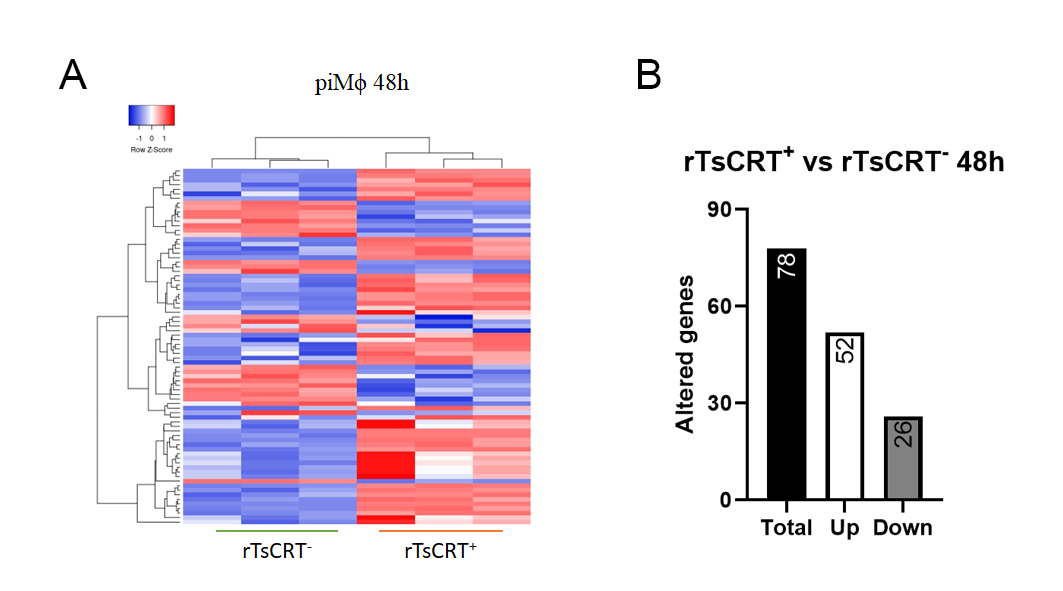

Supplement: SUPPLEMENTARY FIGURE S2 — Time-dependent gene expression of macrophages with persistent infection of hRSV treated with 5 μg of recombinant calreticulin from T. solium (rTsCRT). (A) Heatmap of transcripts altered in piMf by rTsCRT treatment at 48 h. (B) Number of mRNAs deregulated in piMf treated with rTsCRT at 48 h. piMϕ, persistently infected macrophages with hRSV; hRSV, human respiratory syncytial virus. [file Image_2.TIF]

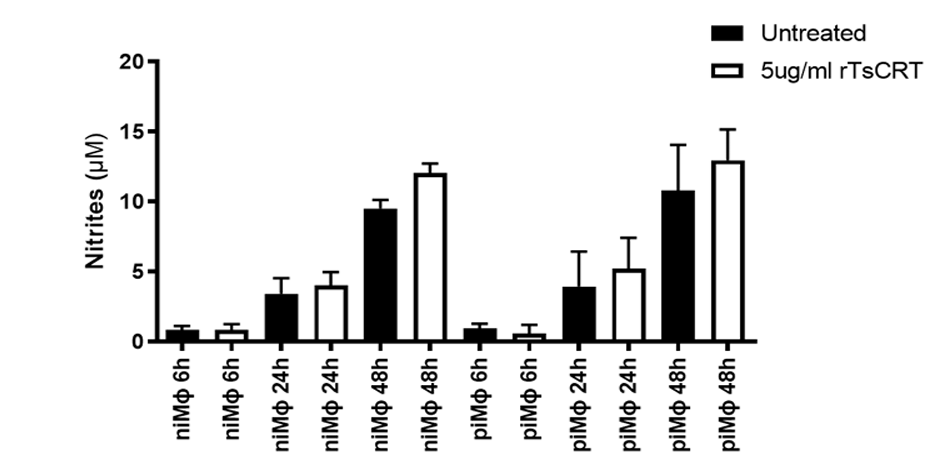

Supplement: SUPPLEMENTARY FIGURE S3 — Production of nitrites in niMϕ and piMϕ after treatment with rTsCRT for 6, 24, and 48 h. rTsCRT, recombinant T. solium calreticulin; niMϕ, non-infected macrophages; piMϕ, persistently infected macrophages with hRSV; hRSV, human respiratory syncytial virus. p-values were calculated by the Mann-Whitney test between niMϕ or piMϕ treated with rTsCRT and their respective control group, none showed statistical significance. [file Image_3.TIF]
