# Supplementary material for: Transcriptome profiling of macrophages persistently infected with human respiratory syncytial virus and effect of recombinant Taenia solium calreticulin on immune-related genes
Source: Front Microbiol. 2024 Sep 4;15:1402589. doi: 10.3389/fmicb.2024.1402589 (PMC11408361; doi:10.3389/fmicb.2024.1402589)
Supplement: Supplementary file 4 [file Table_1.DOCX]

Supplementary Table 1. Changes in gene transcription after 24 h of acute hRSV infection in mouse macrophages and epithelial A549 cells compared with piMφ cultured in the present study.

|  | mRNA expression | | | |
| --- | --- | --- | --- | --- |
|  | Mouse Mφ | | Human A549 cells | piM |
| Gene  Ref. | *Makris et al.*  *2016* | *Ravi et al. 2013* | *Ampuero et al.*  *2018* | *Current results* |
|  |  |  |  |  |
| Il6 | ↑ | ↑ | ↑ | ↑ |
| Il1-α | ↑ | ↑ | ↑ | ↑ |
| Il1-β | ↑ | ↑ | – | ↑ |
| Il2 | – | – | – | – |
| Il4 | – | – | – | – |
| Il8 | n.e. | – | – | – |
| Il5 | – | – | – | – |
| Il10 | – | ↑ | – | – |
| Il12p35 | – | – | – | ↑ |
| Il13 | – | – | – | – |
| Il17 | – | – | – | – |
| Il18 | – | ↑ | ↑ | ↓ |
| Cxcl10 | ↑ | ↑ | – | ↑ |
| Cxcl11 | n.e. | – | ↑ | – |
| Cxcl1 | ↑ | ↑ | ↑ | – |
| Cxcl9 | ↑ | – | – | – |
| Ccl3 | ↑ | ↑ | – | – |
| Ccl2 | ↑ | ↑ | – | – |
| Ddx58/Rig1 | n.e. | ↑ | ↑ | ↓ |
| Ifn-γ | – | – | – | – |
| Tnf | ↑ | ↑ | – | ↓ |
| Rsad2 | ↑ | ↑ | – | ↑ |
| Pkr | ↑ | ↑ | ↓ | ↓ |
| Oas | ↑ | ↑ | ↑ | ↓ |
| Isg15 | n.e. | ↑ | ↑ | – |
| Mx1 | n.e. | ↑ | ↑ | ↓ |
| Ifi44 | n.e. | ↑ | ↑ | – |
| Ifit1/Isg56 | n.e. | ↑ | ↑ | ↑ |
| Ifit2/Isg54 | n.e. | ↑ | – | ↑ |
| Ifit3/Isg60 | n.e. | ↑ | ↑ | – |
| Ifih1 | n.e. | ↑ | ↑ | – |
| Oasl | n.e. | ↑ | ↑ | – |
| Stat1 | n.e. | ↑ | ↑ | ↓ |
| Stat2 | n.e. | ↑ | ↑ | ↓ |
| Irf9 | n.e. | ↑ | ↑ | ↓ |
| Method | PCR | Microarray | Microarray | Microarray |
